# No Influence of *Indy* on Lifespan in *Drosophila* after Correction for Genetic and Cytoplasmic Background Effects

**DOI:** 10.1371/journal.pgen.0030095

**Published:** 2007-06-15

**Authors:** Janne M Toivonen, Glenda A Walker, Pedro Martinez-Diaz, Ivana Bjedov, Yasmine Driege, Howard T Jacobs, David Gems, Linda Partridge

**Affiliations:** 1 Department of Biology, University College London, London, United Kingdom; 2 Institute of Medical Technology and Tampere University Hospital, FI-33014, University of Tampere, Tampere, Finland; Stanford University School of Medicine, United States of America

## Abstract

To investigate whether alterations in mitochondrial metabolism affect longevity in *Drosophila melanogaster,* we studied lifespan in various single gene mutants, using inbred and outbred genetic backgrounds. As positive controls we included the two most intensively studied mutants of *Indy,* which encodes a *Drosophila* Krebs cycle intermediate transporter. It has been reported that flies heterozygous for these *Indy* mutations, which lie outside the coding region, show almost a doubling of lifespan. We report that only one of the two mutants lowers mRNA levels, implying that the lifespan extension observed is not attributable to the *Indy* mutations themselves. Moreover, neither *Indy* mutation extended lifespan in female flies in any genetic background tested. In the original genetic background, only the *Indy* mutation associated with altered RNA expression extended lifespan in male flies. However, this effect was abolished by backcrossing into standard outbred genetic backgrounds, and was associated with an unidentified locus on the X chromosome. The original *Indy* line with long-lived males is infected by the cytoplasmic symbiont *Wolbachia*, and the longevity of *Indy* males disappeared after tetracycline clearance of this endosymbiont. These findings underscore the critical importance of standardisation of genetic background and of cytoplasm in genetic studies of lifespan, and show that the lifespan extension previously claimed for *Indy* mutants was entirely attributable to confounding variation from these two sources. In addition, we saw no effects on lifespan of expression knockdown of the *Indy* orthologues *nac-2* and *nac-3* in the nematode Caenorhabditis elegans.

## Author Summary

Human life expectancy is increasing in many populations. Research on aging has gained great attention recently by discoveries of mutations that slow down aging in relatively short-lived models. Studies carried out in yeast, worms, and flies have revealed evolutionarily conserved mechanisms of aging, which are therefore likely to be relevant to mammals, including humans. Therefore, they can provide an important stepping stone for more time-consuming and expensive experiments on mammals. Lifespan studies can be complicated by interactions of genes under study with the environment and with other genes. These effects can be substantially larger than the effects of some mutations with a bona fide effect on lifespan. Here, the authors studied aging in fruit flies using previously described long-lived mutants in the gene *Indy,* as positive controls for other experiments. Surprisingly, they discovered that *Indy* mutations do not increase lifespan when the genetic background effects are removed. Similarly, knockdown of genes with a similar function in worms do not increase lifespan in this study. The work presented provides an illustration of how genetic background, and possibly the presence of endosymbionts, can confound studies of the genetics of aging and lead to the spurious appearance of single gene effects on aging where none in fact exist.

## Introduction

Mutations in single genes in invertebrate model organisms have been used with great success to discover developmental mechanisms that are evolutionarily conserved in mammals. More recently, it has become apparent that the aging process, too, can be investigated by analysis of single gene mutations that extend lifespan. Thanks in particular to their short lifespans, yeast, nematode worms (C. elegans) and fruit flies (D. melanogaster) have revealed signalling pathways that modulate aging in multiple species. These include the insulin/IGF-like signalling pathway [1–5], the amino-acid-sensing target of rapamycin (TOR) pathway [6–8], and the stress-responsive JNK pathway [9–11].

Typically, the mutations used to study developmental mechanisms cause robust phenotypes that are expressed in a range of genetic backgrounds. Moreover, they are not greatly affected by environmental variation, at least not within the range normally encountered during laboratory studies. By contrast, lifespan is highly sensitive to genetic background and environment, necessitating careful precautions when trying to attribute an increase in lifespan to the effects of a single gene mutation. Natural and laboratory populations of outbred, diploid organisms, such as *Drosophila* and mice, can harbor substantial quantitative genetic variation for lifespan [12–16], and different wild-type strains can therefore differ considerably in longevity. In addition, as is often the case for fitness-related traits, longevity is shortened by inbreeding depression, and increased by heterosis when separate inbred strains are crossed with each other [17]. Use of inbred laboratory strains in aging research is risky, because fixation of deleterious alleles in such stocks can result in identification of alleles that extend lifespan merely by suppressing shortened lifespan in a strain-specific manner [18,19]. For these reasons, when examining the effects of single gene mutations on lifespan it is preferable to backcross into an outbred genetic background with a full, healthy lifespan, similar to that of wild-caught *Drosophila*.

Mutations in single genes can also interact epistatically with the genetic background used and such interactions can be complex and sometimes sex-specific [19–21]. Furthermore, laboratory culture, with its abundant and accessible food supply and pressure for rapid and copious reproduction, can lead to the evolution of accelerated sexual maturation, elevated fecundity, and shorter lifespan [18,22–24]. As in inbred strains, a mutation may, potentially, increase lifespan by reversing the lifespan-shortening effects of adaptation to laboratory conditions. Thus, it is important to analyse putative aging genes in several genetic backgrounds with healthy lifespans. An additional confounding factor, almost routinely ignored in aging studies, is maternally inherited *Wolbachia,* an intracellular symbiotic bacterium that can have unpredictable effects on host fitness–related traits, including lifespan [25–29]. Widespread infection of *Wolbachia* within laboratory stocks has been shown in a recent survey, indicating its presence in approximately 30% of stocks currently housed at the Bloomington *Drosophila* Stock Center [30].

We tested the effects on lifespan of heterozygous, single gene mutations affecting the mitochondrial translation machinery and nucleotide metabolism. We were encouraged to pursue this direction by our preliminary finding that flies heterozygous for a mutation in a mitochondrial ribosomal protein S12 (encoded by *technical knockout*, *tko*) were longer-lived than wild-type flies, without obvious defects in growth or developmental time. As a positive control for these experiments, two mutants for *Indy* (*I'm not dead yet*) were used. Both *Indy^206^* and *Indy^302^* alleles have been reported to result in very long-lived flies in the heterozygous state, and to a lesser extent in homozygotes [31]. *Indy* encodes a plasma membrane Krebs cycle intermediate transporter [32] and *Indy* mutants are reported to cause decreased expression of the gene product [31,33, and references therein]. This strong heterozygous phenotype suggests that mild reduction in expression of *Indy* has a large impact on lifespan without reduction in the rate of development or growth. Thus, the *Indy* mutants were potentially similar to heterozygous mutations affecting mitochondrial translation machinery in terms of their lack of developmental or physiological phenotypes coupled with extended adult lifespan.

Instead, we discovered that in an outbred genetic background *tko* and other mitochondrial mutations studied had no effect on lifespan and, surprisingly, neither did either *Indy* allele in most backgrounds tested. More specifically, we found that *Indy^302^* did not extend lifespan in either sex in any genetic background, while *Indy^206^* was associated with increased lifespan only in one of three genetic backgrounds studied, and even then the effect was male-specific. This genetic-background-specific extension of lifespan in males was largely abolished by tetracycline (TET) treatment, which also removed the intracellular symbiont *Wolbachia* from this mutant stock. The apparent effect of *Indy^206^* on lifespan was thus in large part attributable to the presence of a TET-sensitive modifier. Furthermore, the residual lifespan extension observed was fully reproduced by introducing Chromosome X (but not the *Indy^206^* mutation on Chromosome 3) from the long-lived line into a new genetic background. The *Indy^206^* mutation itself thus played no role in the extension of lifespan. Additionally, three independent RNAi-knockdown experiments targeting worm orthologues of *Indy*, *nac-2* and *nac-3*, also implicated in extended lifespan by previous studies [34,35], did not extend lifespan in our hands.

## Results

### Effects of Mitochondrial Mutations on Lifespan

In *C. elegans,* mutation or knockdown of several genes encoding proteins in the mitochondrial respiratory chain leads to reduced lifespan [36,37] but of many others instead increases lifespan [38–40], by mechanisms that remain uncertain. We examined heterozygous, single gene mutations in flies, to test whether mild impairment of mitochondrial function can lead to extended lifespan. In a pilot experiment, heterozygosity for *tko^25t^,* a hypomorphic allele of mitochondrial ribosomal protein S12 [41–43] increased median lifespan by 18% (unpublished data). To verify our finding in a standard genetic background, the *tko^25t^* and *sesB^1^* alleles (encoding mitochondrial adenine nucleotide translocase), together with a further candidate mutant, *bonsai^1^,* affecting mitochondrial ribosomal protein S15 [44], were backcrossed into the *white* Dahomey (*w* ^Dah^) background and lifespan of heterozygous virgin females was then measured. Females were tested, because both *tko* and *sesB* are located on the X chromosome and hence adversely affect hemizygous mutant males. Virgins were used to avoid potential confounding effects of the mutations on female reproduction, which could affect lifespan. As a positive control we used *Indy^206^*/+ and *Indy^302^*/+ females, both reported to be long-lived [31]. Both alleles were backcrossed into our laboratory background *w* ^Dah^, as for the mitochondrial mutants.

**Figure 1:**
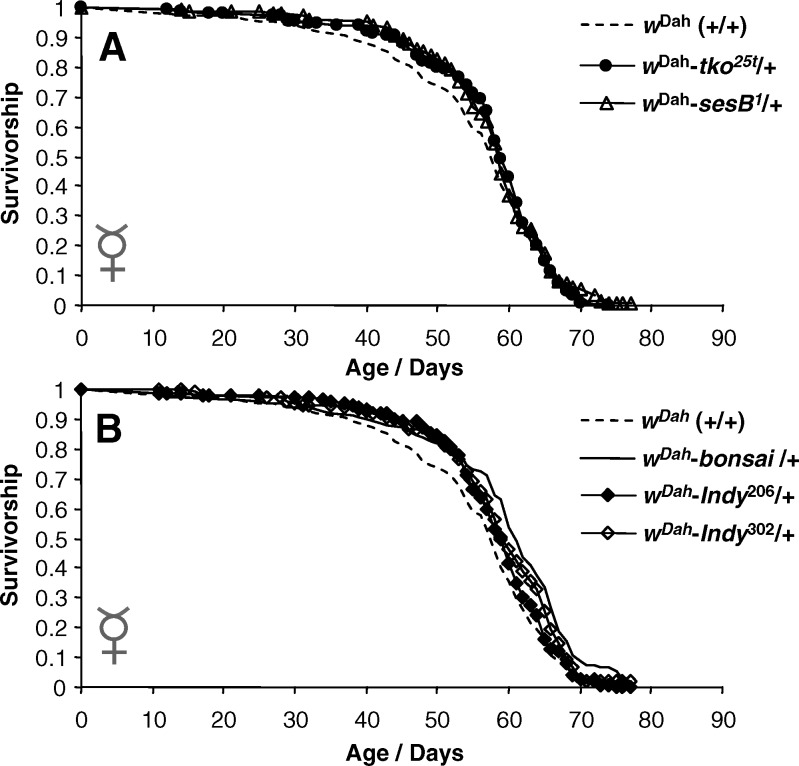
Effects on Longevity of *tko^25t^* and *Indy* Are Abolished by Backcrossing A) Virgin females backcrossed six times to *w* ^Dah^. Median lifespans are 59, 59, and 58 d for *sesB^1^*/+, *tko* ^25t^/+, and *w* ^Dah^ (+/+) females, respectively. Log-Rank test χ^2^ and *p*-values: *sesB^1^*/+ versus +/+ (χ^2^ = 5.59, *p* = 0.0181), *tko* ^25t^/+ versus +/+ (χ^2^ = 1.47, *p* = 0.2258), and *sesB^1^*/+ versus *tko* ^25t^/+ (χ^2^ = 0.84, *p* = 0.3583). B) Virgin females backcrossed six times to *w* ^Dah^. Median lifespans are 60, 60, 62, and 58 d for *Indy^206^*/+, *Indy^302^*/+, *bonsai^1^*/+, and *w* ^Dah^ (+/+) females, respectively. Log-Rank test χ^2^ and *p*-values: *Indy^206^*/+ versus +/+ (χ^2^ = 5.14, *p* = 0.0234), *Indy^302^*/+ versus +/+ (χ^2^ = 9.50, *p* = 0.0021), and *bonsai^1^*/+ versus +/+ (χ^2^ = 31.86, *p* < 0.0001).

When tested after six generations of backcrossing, the longevity phenotype of *tko^25t^*/+ flies had almost disappeared and there was also no significant difference between *tko^25t^*/+ and *sesB^1^*/+ lifespans (Figure 1A). Thus, the increased lifespan seen in the pilot experiment was not attributable to the *tko^25t^* mutation itself, but most likely reflected heterosis (hybrid vigour) between the mutant and the control strain. *bonsai*/+ females (Figure 1B) did show a small but significant increase in median lifespan relative to *w* ^Dah^ (+/+) and *tko^25t^*/+. However, the effect was so small that we chose not to study this further.

### The Effects of *Indy* Mutations on Longevity

To our surprise, the backcrossed *Indy^206^*/+ and *Indy^302^*/+ females were not long-lived either. Instead, their lifespan was intermediate between *w* ^Dah^ and *bonsai*/+ (Figure 1B). We were concerned that long-term maintenance of *Indy* alleles as homozygotes might have dissipated the phenotype, for example, by selection of suppressor mutations. We also wondered whether the discrepancy between our results and earlier reports might reflect differences in the food conditions, or our use of virgin females in the experiment. Recently, strong condition dependency has been reported for another long-lived mutant, *methuselah* [45]. We therefore backcrossed our mutant lines further and investigated the effects of *Indy^206^* and *Indy^302^* on lifespan in more detail, comparing inbred and outbred laboratory genetic backgrounds. In subsequent experiments, we also included the original lines, which had not been further backcrossed, for comparison. In the following text prefixes CS-, *w* ^Dah^-, and *w^1118^*- stand for the original Canton S, *white* Dahomey and *w^1118^* backgrounds, respectively.

The original CS-*Indy^206^,* CS-*Indy^302^,* and the control strain CS-1085 (from the same mutagenesis but with the insert located outside the *Indy* region) were backcrossed for a further 6–10 generations to the outbred *w* ^Dah^ stock, ensuring that cytoplasmic constituents, such as mitochondria, were derived from the *w* ^Dah^ strain (see Materials and Methods). These mutations were also backcrossed into an inbred *w^1118^* stock for five generations, to determine the effects of a different, inbred genetic background.

**Figure 2:**
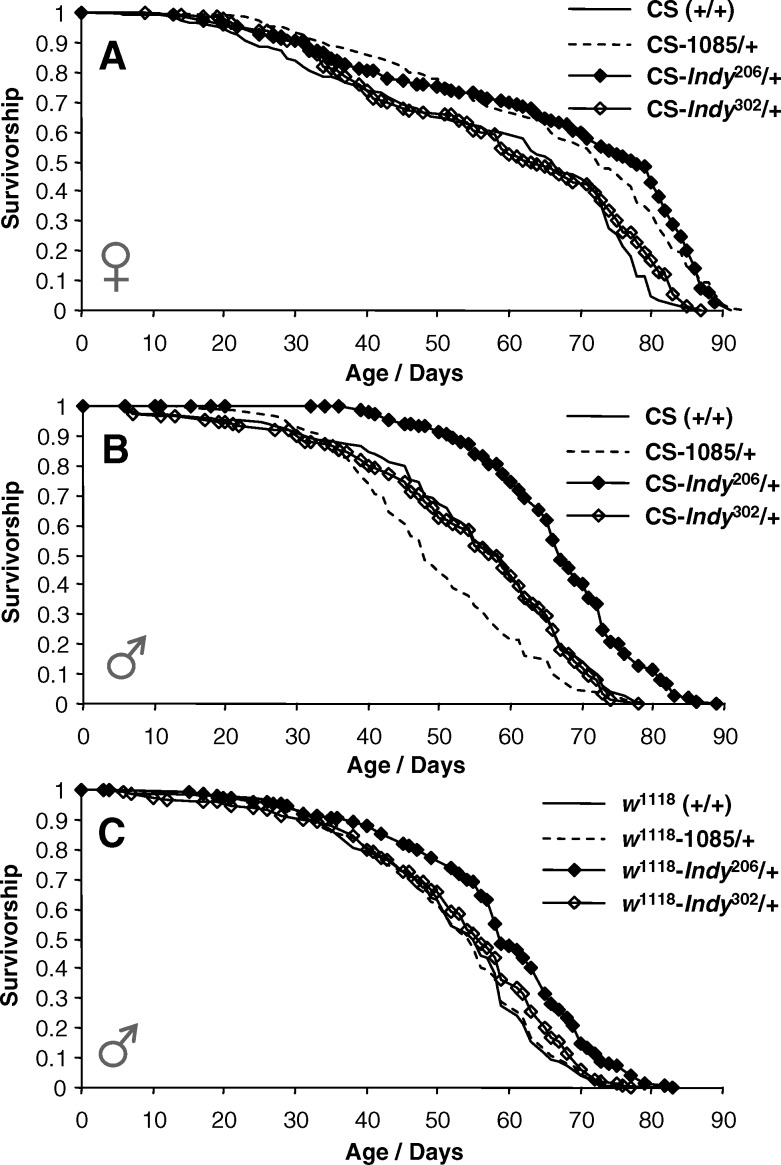
Association of *Indy^206^* Line with Longevity Is Diminished by Moderate Backcrossing. A) Once-mated original females in corn meal food. Median lifespans are 78, 63, 73, and 67 d for CS-*Indy^206^*/+, CS-*Indy^302^*/+, CS-1085/+, and Canton S (+/+) females, respectively. Log-Rank test χ^2^ and *p*-values: CS-*Indy^206^*/+ versus +/+ (χ^2^ = 37.64, *p* < 0.0001), CS-*Indy^206^*/+ versus CS-1085/+ (χ^2^ = 0.69, *p* = 0.4065), CS-*Indy^302^*/+ versus +/+ (χ^2^ = 0.72, *p* = 0.3951), CS-*Indy^302^*/+ versus CS-1085/+ (χ^2^ = 19.97, *p* < 0.0001), CS-*Indy^206^*/+ versus CS-*Indy^302^*/+ (χ^2^ = 35.59, *p* < 0.0001), and +/+ versus CS-1085/+ (χ^2^ = 23.72, *p* < 0.0001). B) Original males in corn meal food. Median lifespans are 67, 48, 58, and 59 d for CS-*Indy^206^*/+, CS-*Indy^302^*/+, CS-1085/+ and Canton S (+/+) males, respectively. Log-Rank test χ^2^ and *p*-values: CS-*Indy^206^*/+ versus +/+ (χ^2^ = 54.82, *p* < 0.0001), CS-*Indy^206^*/+ versus CS-1085/+ (χ^2^ = 132.11, *p* < 0.0001), CS-*Indy^302^*/+ versus +/+ (χ^2^ = 0.33, *p* = 0.5655), CS-*Indy^302^*/+ versus CS-1085/+ (χ^2^ = 13.33, *p* = 0.0003), CS-*Indy^206^*/+ versus CS-*Indy^302^*/+ (χ^2^ = 60.20, *p* < 0.0001), and +/+ versus CS-1085/+ (χ^2^ = 18.33, *p* < 0.0001). C) Males backcrossed for five generations into *w^1118^* (in cornmeal food). Median lifespans are 59, 56, 55, and 55 d for *w^1118^*-*Indy^206^*/+, *w^1118^*-*Indy^302^*/+, *w^1118^*-1085/+, and *w^1118^* (+/+) males, respectively. Log-Rank test χ^2^ and *p-*values: *Indy^206^*/+ versus +/+ (χ^2^ = 24.40, *p* < 0.0001), *Indy^206^*/+ versus 1085/+ (χ^2^ = 22.30, *p* < 0.0001), *Indy^302^*/+ versus +/+ (χ^2^ = 2.34, *p* = 0.1265), *Indy^302^*/+ versus 1085/+ (χ^2^ = 1.74, *p* = 0.1867), *Indy^206^*/+ versus *Indy^302^*/+ (χ^2^ =13.39, *p* = 0.0003), and +/+ versus 1085/+ (χ^2^ = 0.00, *p* = 0.9787).

We first performed further tests to try to reproduce the reported lifespan extension in the original, heterozygous CS-*Indy* lines [31]. To be as faithful as possible to the original methods [31], we used a similar cornmeal-based food medium and we also housed experimental flies in both mixed-sex and once-mated, single-sex conditions. However, similar to our earlier findings with *w* ^Dah^-backcrossed virgin females, we did not see lifespan-extension in the original CS-*Indy^206^*/+ and CS-*Indy^302^*/+ females (Figure 2A). Although we did see a moderate, 16% increase in the median lifespan of CS-*Indy^206^*/+ females compared with CS (+/+), this was not significantly different to the control strain CS-1085/+. Lifespan in CS-*Indy^302^*/+ females was not significantly different from that of CS (+/+), and these flies were shorter lived than both control CS-1085/+ and CS-*Indy^206^*/+ females.

By contrast, we did confirm that CS-*Indy^206^*/+ males are long-lived, and measured a mean lifespan similar to that observed in [31] (Figure 2B; 14% and 40% increase in the median lifespan of CS-*Indy^206^*/+ males relative to CS (+/+) and CS-1085/+ males, respectively). The original CS-*Indy^302^*/+ males were not long-lived compared with CS (+/+), but showed 21% increase in median lifespan compared with CS-1085/+ males. It should be noted also that CS (+/+) males were 23% longer lived than CS-1085/+ males, suggesting that these two control lines are not in a comparable genetic background, or that heterozygosity for the 1085 insertion has an adverse effect on male longevity. The latter is unlikely because, after five generations of backcrossing to the inbred *w^1118^* strain, the w*^1118^*-1085/+ control males behaved identically to the parental *w^1118^* (+/+) line (Figure 2C), median lifespan for both being 55 days. Backcrossed w*^1118^*-*Indy^302^*/+ males also behaved as the controls, showing median lifespan of 56 days. The w*^1118^*-*Indy^206^*/+ mutant males, however, still showed a small 7% median lifespan-extension compared with both controls, the median being 59 days. The results were similar in both once-mated females kept as single sex and females kept in mixed sex groups with males, although mixed sex conditions drastically reduced lifespans of females, regardless of their genotype (unpublished data). These data show that, on cornmeal-based food, using either mixed or separate sex conditions, only one of the mutant alleles under study resulted in increased lifespan, and only in males.

### The Effects of *Indy* Mutations on Gene Expression

**Figure 3:**
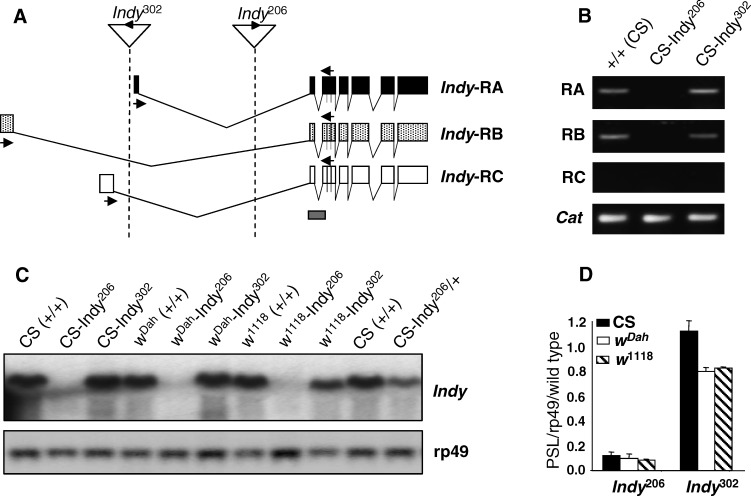
Alternative Transcripts and Gene Expression in *Indy* Mutants A) *Indy* encodes three putative transcripts (RA, RB, and RC) that differ in their ultimate 5′-exons. The insertion sites and orientations of *Indy^206^* and *Indy^302^* are shown (triangles and dashed line). The isoform-specific upstream primers (IndyRA-51, IndyRB-51, and IndyRC-51) and a downstream common region primer (IndyR-31) are indicated as arrows. Common region probe used for northern analysis (C) is indicated as grey box. B) cDNAs from the homozygous *Indy* mutants and CS (+/+) control analyzed by isoform-specific PCR. Expression of all isoforms was abolished by *Indy^206^* mutation. *Indy^302^* line expressed both isoforms present in the wild-type cDNA. There was no evidence for isoform RC whereas the control *(Catalase)* could be amplified from all cDNA samples. C) Expression analysis of *Indy* mutants in different genetic backgrounds. Northern hybridization from wild type (+/+), *Indy^206^,* and *Indy^302^* homozygotes in CS, *w* ^Dah^, and *w^1118^* genetic background. The two last lanes show CS (+/+) compared to intermediate *Indy^206^*/+ heterozygous expression. Ribosomal protein rp49 hybridization is shown for loading. D) Phosphorimager quantification of the northern data. The graph shows mean (and standard error) of two separate hybridizations normalized by rp49 and is shown as relative ^32^P-stimulated luminescence compared with CS, *w* ^Dah^, or *w^1118^* flies.

The lack of phenotype in *Indy* flies was surprising, and we therefore confirmed that the *Indy* mutations were still present in our stocks. The mutations were as published [31] and were identical in the three genetic backgrounds (see Figure S1). We were particularly interested in why, even in the original genetic background, we could confirm the reported lifespan extension in *Indy^206^* males but not in *Indy^302^* males. The effect of the different mutant alleles on *Indy* expression has not been shown previously, and we therefore examined the consequences of the two mutations for *Indy* mRNA levels. Based on annotation in FlyBase [46], *Indy* (annotation symbol CG3979) encodes three putative transcripts (*Indy*-RA, Indy-RB, and *Indy*-RC; Figure 3A) that differ only in their 5′-exons. To determine how the *Indy^2^* ^06^ and *Indy^302^* alleles affect the expression of these alternative *Indy* transcripts, we performed PCR with splice variant–specific primers and template cDNA obtained from homozygous *Indy^2^* ^06^ and *Indy^302^* mutants (Figure 3B). *Catalase (Cat)* was amplified as a control for cDNA quality, and also to confirm that its expression is not affected in *Indy* mutants (the *Cat* gene is located proximal to *Indy* in the third chromosome). In tests of wild-type flies, cDNA for variants RA and RB was seen, but not for variant RC. Long-range PCR using genomic DNA as a template confirmed that this was not due to a problem with the function of the primers (unpublished data). All three variants were absent from the *Indy^206^* cDNA sample, consistent with the decreased expression of protein reported in [33]. However, the *Indy^302^* mutants showed a similar expression pattern to the wild type. Because PCR methods in general are only semi-quantitative, we performed a northern hybridization using RNA samples from homozygous *Indy^206^* and *Indy^302^* males (Figure 3C). The result confirmed our finding from the PCR assay, namely, that, whereas the *Indy^206^* mutation had a strong effect on gene expression, *Indy^302^* had no detectable effect. A phosphorimager quantification showed that, whereas expression in the *Indy^206^* lines was less than 10% of the wild-type levels, the expression in *Indy^302^* was typically 85% to 110% compared to the corresponding wild-type strains (Figure 3D).

### Backcrossing Abolishes *Indy^206^*-Related Longevity

As shown in Figures 2B and 2C, the increase in lifespan of *w^1118^*-*Indy^206^*/+ males after five generations of backcrossing was clearly diminished compared with the same mutation in the original genetic background. We therefore investigated whether thorough backcrossing of *Indy^206^* into the outbred *w* ^Dah^ stock would completely abolish the phenotype. The extent to which lifespan is affected by *Indy* mutations might also depend on food type and we therefore repeated the experiment using sugar-and-yeast-based food (SY).

**Figure 4:**
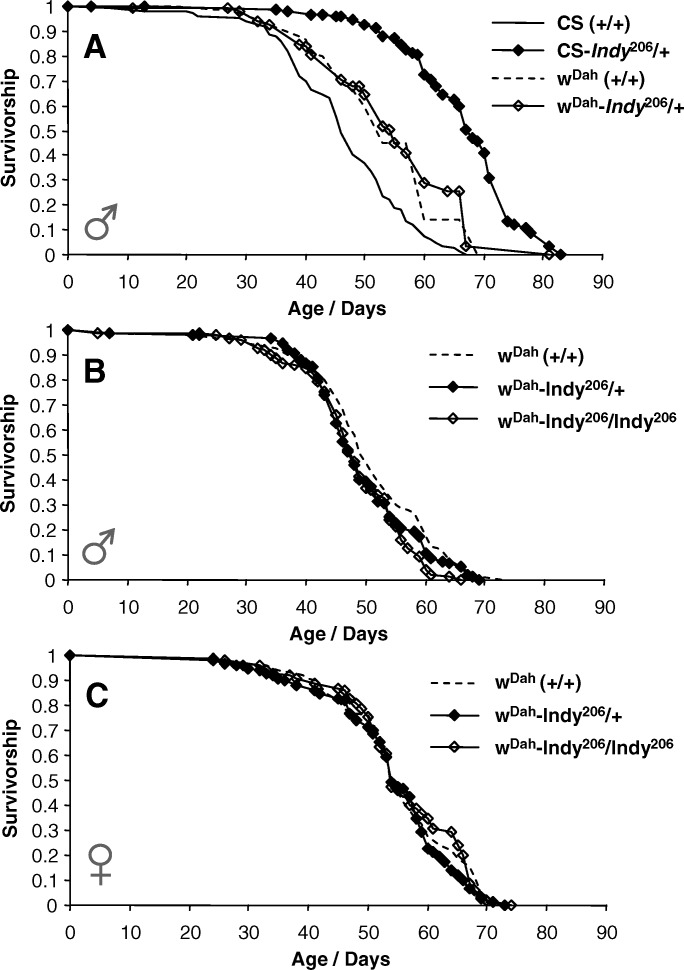
Successive Backcrossing Abolishes *Indy^206^*-Associated Longevity A) Survival of the original versus *w* ^Dah^-backcrossed (×8) males on SY food. The data are male progeny derived from crosses between *Indy^206^* (or +/+) homozygote mothers and +/+ males. Median lifespan is 68 and 46 d for CS-*Indy^206^*/+ and CS (+/+) males, respectively (Log rank test χ2 = 131.17, *p* < 0.0001), and 55 and 53 d for *w* ^Dah^-*Indy^206^*/+ and *w* ^Dah^ (+/+) males, respectively (Log rank test χ2 = 0.06*, p* = 0.8009). B) Survival of the *w* ^Dah^-backcrossed (×10) males on SY food. The flies are all progeny from crosses between *w* ^Dah^-*Indy^206^*/+ females and *w* ^Dah^-*Indy^206^*/+ males. Median lifespans are 48, 48, and 50 d for homozygous, heterozygous and +/+ males, respectively. Log-Rank test χ^2^ and *p-*values: *w* ^Dah^-*Indy^206^*/+ versus *w* ^Dah^-*Indy^206^*/ *Indy^206^* (χ^2^ = 1.34, *p* = 0.2467), *w* ^Dah^-*Indy^206^*/+ versus +/+ (χ^2^ = 2.08, *p* = 0.1493), and *w* ^Dah^-*Indy^206^*/ *Indy^206^* versus +/+ (χ^2^ = 7.37, *p* = 0.0066). C) Survival of *w* ^Dah^-backcrossed (×10) females on SY food. The flies are all progeny from the same crosses as males in Figure 3B. Median lifespans are 54, 54 and 54 d for homozygous, heterozygous and +/+ females, respectively. Log-Rank test χ^2^ and *p-*values: *w* ^Dah^-*Indy^206^*/+ versus *w* ^Dah^-*Indy^206^*/ *Indy^206^* (χ^2^ = 1.37, *p* = 0.2423), *w* ^Dah^-*Indy^206^*/+ versus +/+ (χ^2^ = 0.63, *p* = 0.4272), and *w* ^Dah^-*Indy^206^*/ *Indy^206^* versus +/+ (χ^2^ = 0.00*, p* = 0.9980).

We first measured lifespan of *w* ^Dah^ *-Indy^206^*/+ males, backcrossed for eight generations, using SY food (Figure 4A). The backcrossed *w* ^Dah^ *-Indy^206^*/+ males were not long-lived, and behaved as the *w* ^Dah^ control (median 55 and 53 days, respectively). As a positive control for the effect of the backcrossing we exposed the original, non-backcrossed mutant lines to the same SY food medium. Again, a robust 48% extension was seen in median lifespan in the original CS-*Indy^206^*/+ males compared with CS (+/+) control (median lifespan 68 and 46 days, respectively). The mean lifespan of CS-*Indy^206^*/+ males was again very similar to the published data (mean 66.4 days compared with 71 days in [31]).

We repeated the experiment after ten generations of backcrossing and tested *w* ^Dah^ *-Indy^206^* males and females, in homozygous and heterozygous condition (Figures 4B and 4C). No lifespan extension was observed in either genotype, in males (Figure 4B) or in females (Figure 4C). The same experiment was conducted using *w* ^Dah^ *-Indy^302^* with similar results, except that the females homozygous for the *Indy^302^* insertion were clearly short lived (Figure S2A and S2B). Together, these data confirm that, with SY food as well as with corn meal-based medium (Figure 2B), one may observe the substantial lifespan-extension in the original, non-backcrossed males heterozygous for *Indy^206^*, but that this increase in lifespan is not present in thoroughly backcrossed males carrying the same mutation.

### TET Treatment Diminishes Indy^206^-Related Longevity

**Figure 5:**
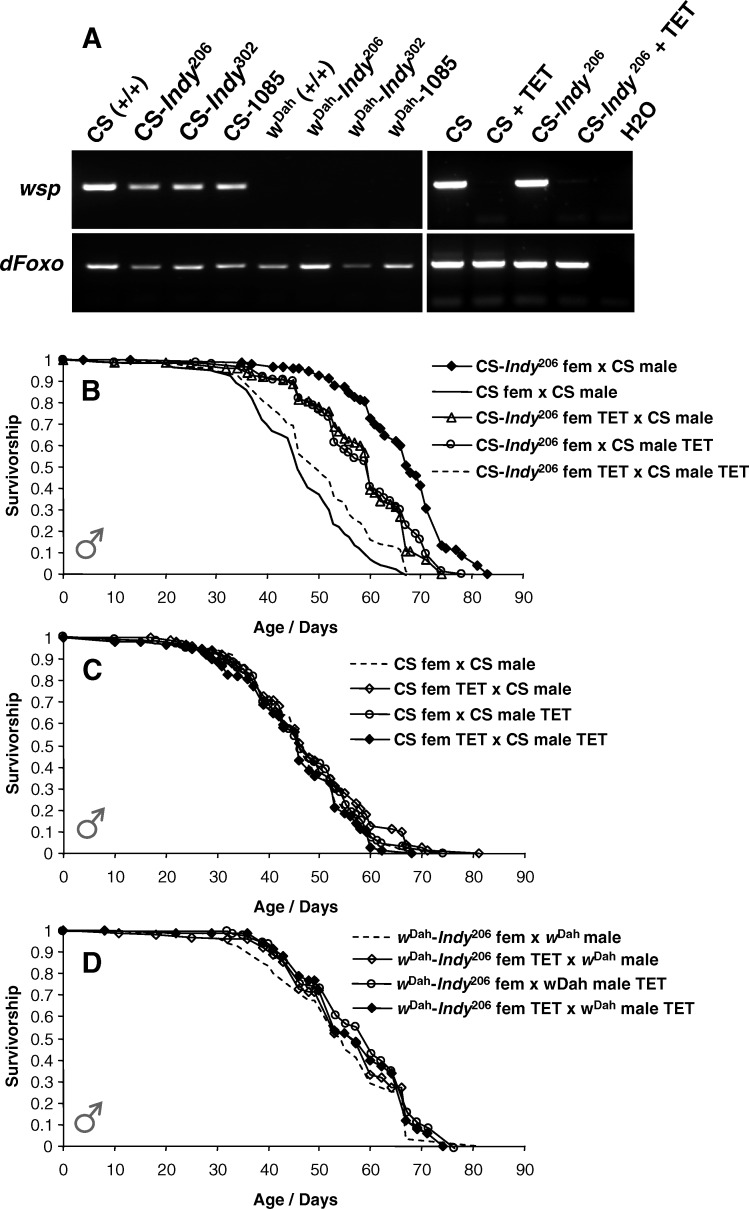
Tetracycline Treatment Greatly Modulates *Indy^206^*-Related Longevity Crosses conducted to obtain male progeny for lifespan experiments are shown next to the symbol keys (mothers left, fathers right). All lifespan experiments were carried out on SY food. A) Detection of *Wolbachia* infection by PCR using primers specific to *Wolbachia* surface protein (*wsp,* upper panels). *dFOXO* was amplified as a control for DNA quality (lower panels). The original Canton S background is infected with *Wolbachia* and this infection is absent in *w* ^Dah^ background (upper left panel). TET treatment removed *Wolbachia* from infected lines (upper right panel). B) TET treatment of parents drastically shortens lifespan of long-lived CS-*Indy^206^*/+ males. All experimental flies are heterozygous for *Indy^206^,* except the CS control. Treatment father or mother alone had an equal effect on lifespan of the progeny (Log-Rank test χ^2^ = 0.31, *p* = 0.5794). When both parents were treated, the CS-*Indy^206^*/+ progeny was slightly but significantly different from the Canton S control (Log-Rank test χ^2^ = 6.86, *p* = 0.0088). All other conditions were significantly different from each other (Log-Rank test, *p* < 0.0001). C) TET treatment of parents has no effect on lifespan of Canton S control males. All experimental males are wild type (CS). There are no statistical differences among any conditions (Log-Rank test, *p* > 0.065). D) Crosses similar to those in (B) were conducted using *w* ^Dah^-*Indy^206^* flies. No significant differences were found (Log-Rank test, *p* > 0.145), except when progeny of nontreated parents were compared with progeny of parents where fathers were TET treated (Log-Rank test χ^2^ = 4.13, *p* = 0.0422). fem, females.

Having established that mutations in *Indy* alleles are not themselves causal for longevity, we explored alternative explanations for the male-specific longevity observed in the original *Indy^206^* line. *Wolbachia,* an intracellular symbiont found frequently in *Drosophila* stocks [30], is a maternally derived factor that can potentially modulate longevity. We investigated the *Wolbachia* status of these lines by PCR detection of the gene for *Wolbachia* surface protein *(wsp)* [47,48]. All the original mutant lines, including the Canton S control, were infected by these α-proteobacteria (Figure 5A, upper left panel). We also analysed the mutants (and wild-type controls) in the two other genetic backgrounds used, and found no signs of infection in either the *w* ^Dah^ (Figure 5A) or *w^1118^* (unpublished data) backgrounds.

To test the possibility that the longevity phenotype in the original CS-*Indy^206^* heterozygotes was *Wolbachia* dependent, we used TET treatment, which removes *Wolbachia* infection. Canton S and CS-*Indy^206^* lines were cured by adding 25 μg/ml TET to the food medium for three generations. *Wolbachia*-negative *w* ^Dah^ and *w* ^Dah^-*Indy^206^* lines were also treated with TET to provide drug treatment controls. After treatment, the fly stocks were cultured for several generations in TET-free medium, and the removal of *Wolbachia* from treated lines was confirmed by PCR (Figure 5A, upper right panel). When both parents were TET treated, the resulting CS-*Indy^206^* male progeny showed only a small increase in lifespan relative to Canton S control flies, (Figure 5B, median lifespan 50 days and 46 days, respectively), although this increase was statistically significant. Treatment of one or the other parent only resulted in intermediate lifespans compared with the situation where both parents were nontreated or treated (Figure 5B, open triangles and open circles). Canton S controls were not affected by the treatment (Figure 5C, all median lifespans between 46 and 48 days), implying that there was no adverse effect of treatment on other aspects of metabolism in these flies, such as mitochondrial function. It also showed that *Wolbachia* removal per se does not affect lifespan of Canton S flies. We performed similar crosses using treated and nontreated *Indy^206^* mutants in the *w* ^Dah^ background (Figure 5D) and did not in general see a significant effect of TET treatment, median lifespan being between 55 and 60 days for all groups. We conclude that at least part of the lifespan extension observed in original *Indy^206^* males is the result of a TET-sensitive modifier, possibly *Wolbachia*. However, because a small effect was seen also when only fathers were treated, we cannot exclude a possibility of another bacterial associate.

### X-Chromosomal Modifier of Longevity in CS-*Indy^206^* Line

**Figure 6:**
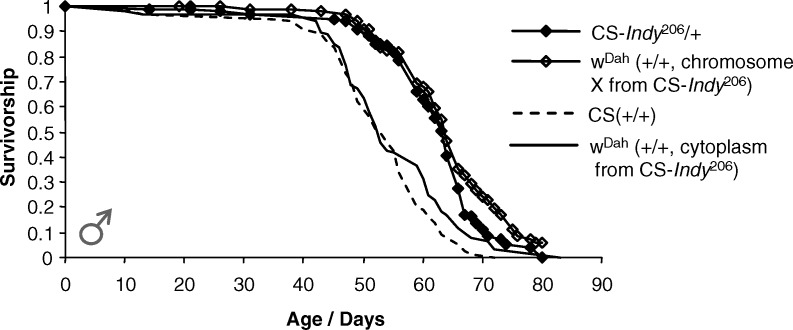
Modifier(s) in the Chromosome X of the CS- *Indy^206^* Mutant Underlies Its Longevity. Lifespan of Canton S (+/+) and long-lived CS- *Indy^206^* males was analyzed on SY food in parallel with lines that no longer contain *Indy^206^* mutation, but still retain Chromosome X or cytoplasmic constituents, including mitochondria and *Wolbachia,* from the CS- *Indy^206^* strain. The strain that only retains cytoplasm from CS- *Indy^206^* (and all nuclear chromosomes from w^Dah^) shows similar lifespan compared with Canton S control (for both, median lifespan 53 d, Log-Rank test χ^2^ = 2.80, *p* = 0.0943). The strain that retains the Chromosome X from CS- *Indy^206^* (and all other chromosomes plus cytoplasm from w^Dah^) shows similar lifespan compared with long-lived CS- *Indy^206^* (for both, median lifespan 64 d, Log-Rank test χ^2^ = 3.76, *p* = 0.0525). All other comparisons between the strains are significant (*p* < 0.0001).

Although the long lifespan of CS-*Indy^206^* males was largely dissipated by TET treatment, it did not completely abolish the phenotype (Figure 5B). We therefore determined the source of this residual effect. Logical possibilities included the mitochondria, and the X chromosome, which in males is maternally derived. We therefore transferred either cytoplasmic constituents or the X chromosome from the long-lived CS-*Indy^206^* strain to the otherwise w^Dah^ genetic background (details in Protocol S1). We took particular care that chromosomes in which recombination between the Canton S and the *w* ^Dah^ chromosomes had potentially occurred were eliminated during the procedure. Importantly, these lines were now wild type with respect to the *Indy* locus. Transfer of cytoplasmic constituents from the long-lived CS-*Indy^206^* to the otherwise *w* ^Dah^ background did not affect longevity (Figure 6, solid line). By contrast, transfer of X chromosome alone from the long-lived CS-*Indy^206^* was enough to extend lifespan of the males in an otherwise *w* ^Dah^ genetic background to match that of the long-lived CS-*Indy^206^* males (Figure 6, open and black diamonds, respectively). This finding demonstrates that the *Indy^206^* mutation itself did not produce the lifespan extension associated with the nuclear genotype of the original CS-*Indy^206^* line. The lifespan extension was due to a combination of a TET-responsive factor together with an X-chromosomal modifier of lifespan in the stock.

### *Indy* Homologs and Aging in C. elegans

In the nematode *C. elegans*, there are three proteins with homology to *Drosophila* INDY. These are NAC-1 (F31F6.6, previously known as ceNAC-1 and ceNaDC1), NAC-2 (R107.1, previously known as ceNAC-2 and ceNaDC3), and NAC-3 (K08E5.2, previously known as ceNAC-3 and ceNaDC2) [34,35]. Previously, the reported influence of *Indy* on lifespan in *Drosophila* [31] motivated tests for similar effects on lifespan of *nac-1, −2,* and *-3* on lifespan in C. elegans. RNA-mediated interference (RNAi) knockdown of *nac-2* [35] and *nac-3* [34] were reported to extend mean lifespan by 19% and 15%, respectively.

**Figure 7:**
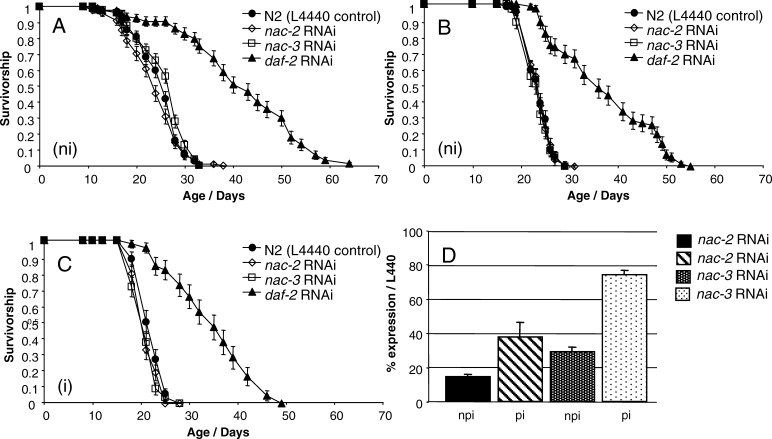
Effects on Lifespan in *C. elegans* of RNAi of *nac-2*, *nac-3,* and *daf-2* (22 °C) Lifespan analysis of the mutants subjected to bacteria-mediated RNAi in nonpreinduced and preinduced conditions (see text for details). N2 (wild-type) worms fed with L440 plasmid was used as a vector control. Three independent replicates of the experiments are shown. Log-Rank test χ^2^ and *p-*values: A) L4440 versus *nac-2* (χ^2^ = 1.82, *p* = 0.1772), L4440 versus *nac-3* (χ^2^ = 4.01, *p* = 0.0452), and L4440 versus *daf-2* (χ^2^ = 137.99, *p* < 0.0001). B) L4440 versus *nac-2* (χ^2^ = 1.02, *p* = 0.8923), L4440 versus *nac-3* (χ^2^ = 0.52, *p* = 0.4727), and L4440 versus *daf-2* (χ^2^ = 39.11, *p* <0.0001). C) L4440 versus *nac-2* (χ^2^ = 3.82, *p* = 0.0507), L4440 versus *nac-3* (χ^2^ = 5.09, *p* = 0.024), and L4440 versus *daf-2* (χ^2^ = 65.76, *p* <0.0001). D) The efficiency of the RNAi of *nac-2* and *nac-3* by the two methods of induction used for lifespan experiments (RT-PCR). On average, *nac-2* was decreased by 85% with nonpreinduced method (npi) or by 62% with preinduced method. *Nac-3* was decreased by 71% nonpreinduced or 30% preinduced. Each bar represents average (and standard error) from two measurements from independent RNA extractions. npi, nonpreinduced; pi, preinduced.

Our negative results regarding the influence of *Indy* on *Drosophila* lifespan motivated us to verify the effects of RNAi knockdown of *nac-2* and *nac-3* on C. elegans lifespan, employing the previously used *nac-2* and *nac-3* RNAi feeding plasmids, kindly provided by Dr You-Yun Fei. Using experimental conditions similar, but not identical, to those in the previous studies (see Materials and Methods) we saw no effect of *nac-2* or *nac-3* RNAi on lifespan in two separate experiments (Figure 7A and 7B). These results could imply that any effects on lifespan of RNAi knockdown of *nac-2* and *nac-3* are sensitive to small differences in experimental conditions. Therefore, we repeated the experiment a third time using conditions more closely replicating the original studies by Fei et al. [34,35], in that RNAi feeding bacteria were preinduced using IPTG before being added to IPTG-containing agar plates. Again, no increases in lifespan were seen (Figure 7C). We verified the efficiency of the RNAi procedure in three ways. First, we used semi-quantitative RT-PCR to check that *nac-2* and *nac-3* mRNA levels were reduced, and they were (Figure 7D). Second, we performed positive control tests in each trial using *daf-2* RNAi. This resulted in a large increase in lifespan in all repeats of the experiment, demonstrating that our RNAi methodology was working normally (Figure 7A–C). Third, we verified by DNA sequencing the identity of the inserts in the *nac-2* and *nac-3* feeding vectors (unpublished data). The results of the three lifespan experiments are summarized in Table 1.

**Table 1:**
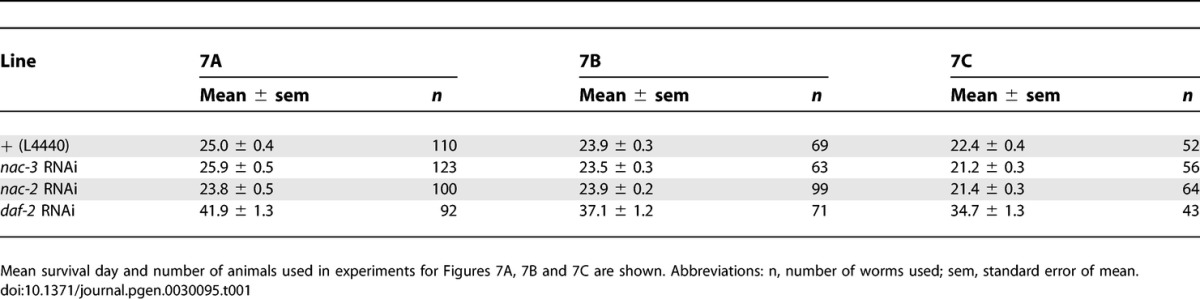
Summary of the C. elegans Lifespan Experiments

## Discussion

The original aim of this study was to establish whether mild mitochondrial defects could extend lifespan in flies, as they do in worms. Here, our findings were inconclusive. As in worms, increases in lifespan resulting from mitochondrial defects might depend largely on the level of electron transport chain inhibition. Alternative approaches to analyse mitochondrial mutations, such as RNAi inhibition of the mitochondrial translation machinery, would be a good way to explore this possibility. For practical reasons we worked with virgin females, and cannot exclude the possibility that virginity could have affected the outcome of our studies. Work with these mutants provides an illustration of how genetic background can be a major determinant of longevity associated with single gene mutations. However, our major and unexpected finding was that the *Indy* mutations, which we had intended to use as positive controls, do not increase lifespan. Instead, treatment with TET abolished much of the original lifespan extension associated with the CS-*Indy^206^* line and substantial lifespan extension was brought about by transfer of X chromosome from the original CS-*Indy^206^* line to a novel genetic background.

### Reduced *Indy* Expression Does Not Confer Longevity

We have shown that two *Indy* mutations, *Indy^206^* and *Indy^302^,* previously reported to extend lifespan to a similar extent, do not decrease expression of *Indy* mRNA to the same extent, and that *Indy^302^* does not decrease it at all (Figure 3). In all three genetic backgrounds tested, the expression of all *Indy* transcripts was severely affected in the *Indy^206^,* but not in the *Indy^302^* mutant. A decrease in transcript levels was reported in both *Indy^206^* and *Indy^302^* mutants ([33] referred therein as unpublished data). Our stocks were verified to be authentic by two independent methods (see Figure S1) and, therefore, we are unable to explain the discrepancy in the results. The data also suggest that only two of the three transcript variants annotated in FlyBase [46] are expressed in adult flies. However, we cannot exclude the possibility of tissue-specific or conditional regulation for the third alternative transcript. When the expression data and lifespan experiments are taken together, inhibition of *Indy* transcription lacks correlation with lifespan extension.

### *Indy* Mutants Are Not Consistently Long Lived

Small, absent, or inconsistent effects of *Indy* alleles on lifespan were reported earlier. When freshly isogenised mutants were tested, only a small lifespan extension was observed in heterozygous *Indy* females in short-lived lines with a genetic background expressing a lethal toxin coupled to an age-dependent molecular biomarker [49]. *Indy^206^* and *Indy^302^* insertions that contain a *lacZ* reporter gene were used as markers to study temporal patterns of gene expression, and their lifespan was reported to be similar to the controls [50,51]. A recent study by Khazaeli et al. [52] could not confirm longevity in males homozygous for *Indy^206^* and *Indy^302^* mutations, although even the homozygous *Indy* mutants were reported to outlive the controls by 10%–20% [31]. Aging-related decline in performance, measured as negative geotaxis, progressed much more rapidly in *Indy* mutants when compared with *chico^1^,* a long-lived mutant of the insulin/IGF-like signalling pathway [53]. When measured as absolute rate of functional decline, *Indy^206^* mutants were not statistically different from wild-type controls [54]. Unlike many other single gene mutations found to extend lifespan, longevity of *Indy* mutants has not been studied in multiple genetic backgrounds before and, even in the original backgrounds, the published results proved difficult to repeat in another laboratory [52].

The lack of longevity that we observed in flies carrying *Indy* mutations was unexpected, because lifespan extensions of 40%–80% were reported in three genetic backgrounds in addition to Canton S [31]. It is not clear, however, whether these findings are derived from thoroughly backcrossed flies or whether F1 hybrids were studied. Based on our results, it seems likely that heterosis between the experimental strains and modifier loci elsewhere in the genome (such as the one described here) account for the lifespan extension seen. The fact that excision of the P-elements from the *Indy* locus apparently rescued longevity [31] might in fact reflect segregation of undefined lifespan-extending modifier(s) in the mutant genetic background, or perhaps loss of *Wolbachia*. Unfortunately the original P-element excision lines are not available for further analysis. Genetic bottlenecks that accompany P-element excisions, or isogenization procedures that result in the introduction of extraneous genetic material, could result in alterations in lifespan. As reported here, the original data on *Indy*-related longevity can be explained by lifespan-modifying elements that are unconnected to the *Indy* mutations themselves. Our results imply that a large part of the lifespan-extending effect is due to an X-chromosomal modifier(s). The fact that longevity determinant(s) transferred with the X chromosome can increase lifespan in an otherwise *w* ^Dah^ genetic background also implies that lack of longevity is not due to “insensitivity” of this background to the levels of Indy, which could potentially result from strain-specific polymorphisms. We have clearly established that *w* ^Dah^ can exhibit similar longevity compared with the original mutant line (see Figure 6), provided that right modifiers are present.

### Genetic Background and Nucleo–Cytoplasmic Interactions

Variation in the nuclear background can strongly influence the extent of longevity resulting from single gene interventions, the best studied examples being manipulations of Cu/Zn-superoxide dismutase expression in adult flies [19,20]. These studies provided evidence that the impact of Cu/Zn-superoxide dismutase overexpression on longevity is generally stronger in short-lived laboratory lines, and that alleles at other loci interact epistatically with the Cu/Zn-superoxide dismutase transgene to modify its ability to extend longevity.

Any particular genetic background is not only defined by its nuclear genome, but also contains a maternally inherited cytoplasmic genome, the mitochondrial DNA. Experiments that combined mitochondrial and nuclear genomes of separate origin have shown that substantial variation in longevity can be attributable to nuclear–mitochondrial interactions [55]. The cytoplasmic endosymbiont *Wolbachia*, like other bacteria, is sensitive to the TET class of antibiotics, and the presence or absence of *Wolbachia* can contribute substantially to variation in longevity [28]. However, as mentioned above, not all *Wolbachia*-positive lines show altered longevity in response to TET treatment ([26,28], see also Figure 5C). We have shown here a decrease in lifespan by TET treatment. This effect was specific for the original long-lived CS-*Indy^206^* line and hence, in this line, the presence of *Wolbachia* was positively associated with longevity. Transfer of cytoplasmic constituents (including mitochondria and *Wolbachia*) to another genetic background, however, did not result in extended lifespan (Figure 6). Similarly, TET treatment of fathers also had a significant effect on lifespan of the male progeny. This implies that the effect of *Wolbachia* is dependent on, and interacts with, other factors in the host genome. We cannot exclude the possibility that the phenotype is dependent on some other bacterial associate in the CS-*Indy^206^* line, which would be similarly eliminated by the drug treatment. However, the fact that *Wolbachia* frequently infects tissues implicated in determination of longevity, such as nerves, fat body, and the ovary [30], is a confounding factor in the genetic analysis of longevity, and deserves more attention in the experimental design.

### *Indy* and Diet

Variation in environmental conditions in which lifespan experiments are conducted can result in problems with reproducibility of published data from different laboratories. For example, differences in mating status due to different housing conditions (mixed sex or single sex) can strongly affect lifespan. One major source of variation that could be especially important with respect to *Indy* is diet, given the role of this gene in nutrient transport. We reproduced, in two very different food types, a robust lifespan extension for the original *Indy^206^* line that had not been further backcrossed. This implies that the effects on lifespan in this line are not highly condition dependent with respect to food type. The best-studied environmental intervention that leads to extended lifespan is dietary restriction (reviewed in [56,57]). Mutations reducing the levels of Indy have been suggested to alter the metabolism of the fly in a way that favours lifespan extension, possibly by inducing a state similar to dietary restriction [31,33,34]. To date, however, no reports have addressed the question of how *Indy* mutations affect survival when dietary conditions are altered. It is also not clear whether long-lived *Indy* mutants impinge upon any downstream effects on other molecules possibly involved in the dietary restriction pathway, such as Sir2 or Rpd3 [58,59]. In our hands, the lifespan of backcrossed *Indy* mutants proved to be the same as wild type over a wide range of food dilutions (PM, unpublished data), implying that *Indy* plays no role in the response to dietary restriction in *Drosophila*.

### *Indy* Homologs and Lifespan in C. elegans

In C. elegans, three gene products showing significant amino-acid sequence homology with *Drosophila* INDY can be found. RNAi knockdown of two of these genes, *nac-2* [35] and *nac-3* [34], has been reported to result in moderate increases in lifespan. By contrast, we saw no effects of RNAi of *nac-2* or *nac-3* RNAi on lifespan, using similar conditions. This could reflect small differences in the RNAi conditions used: for some genes, the effects of RNAi on lifespan are sensitive to small differences in conditions. In this context, it is worth noting that we did not see a marked decrease in body size in animals subjected to *nac-2* RNAi, in contrast to an earlier study [35]. This suggests that RNAi conditions might have been milder in our study, although it is worth emphasizing that *daf-2* RNAi increased lifespan to a degree that is comparable to other studies. We also showed that the conditions that we used were sufficient to substantially reduce *nac-2* and *nac-3* mRNA levels. The basis of the apparent condition dependency of effects of *nac-2* and *nac-3* RNAi C. elegans lifespan will require further elucidation.

### Conclusions

Studies of the genetics of aging in *Drosophila* are highly vulnerable to confounding effects, especially due to heterogeneity between mutant and control populations. Here, we have shown a case in point, based on the analysis of our own initially promising results together with a prominent case from the literature. The data presented here show that mutations in the *Indy* gene do not extend lifespan, and highlight the importance of carefully controlling genetic background in studies of longevity. Standardisation of genetic background can be achieved by successive backcrossing of a putative aging gene, preferably into several healthy, outbred genetic backgrounds with relatively long-lived wild types. The backcrossing must be conducted in a way that ensures passage of cytoplasmic factors to the progeny, and checks should be made for the presence of intracellular endosymbionts such as *Wolbachia*.

## Materials and Methods

### Fly stocks and husbandry.

*tko^25t^* and *sesB^1^* mutant flies were supplied by K. M. C. O'Dell and C.-F. Wu. *bonsai^1^* stock was a kind gift from Mireille Galloni. The wild-type stock Dahomey was collected in 1970 in Dahomey (now Benin) and has since been maintained in large population cages with overlapping generations on a 12L:12D cycle at 25 °C. This method of husbandry maintains lifespan and fecundity at levels similar to freshly caught stocks [24]. The *white* Dahomey (*w* ^Dah^) stock was derived by incorporation of *w^1118^* deletion into the outbred Dahomey background by successive backcrossing. The inbred *w^1118^* background, obtained from the *Drosophila* Stock Center (http://flystocks.bio.indiana.edu), was used in some experiments in parallel with *w* ^Dah^. *Indy* mutant alleles are originally derived from the same mutagenesis, where an effort was made to standardise the genetic background to that of Canton S containing the *w^1118^* deletion [31,60]. The original materials (*Indy^206^* and *Indy^302^* and the control line 1085) were provided by Stephen Helfand to the Institute of Medical Technology in Finland in May 2002, where they were backcrossed for further studies. To backcross these mutants into other genetic backgrounds, females from *w* ^Dah^ or *w^1118^* stocks were first mated with *Indy^206^, Indy^302^,* or 1085 males, to ensure the transfer of cytoplasmic constituents from *w* ^Dah^ or *w^1118^* to the progeny. Heterozygous mutant females were then backcrossed to males with these genetic backgrounds five *(w^1118^)* or ten (*w* ^Dah^) times. The original and backcrossed stocks were maintained in large numbers in culture bottles at 18 °C on a 12L:12D cycle. Ingredients of different food media are described in Protocol S1.

### Drosophila lifespan experiments.

Unless otherwise stated, to obtain heterozygous experimental flies, homozygous mutant fe-males were crossed to corresponding wild-type (Canton S, *w* ^Dah^, or *w^1118^*) males. In one experiment (data in Figure 4B and 4C), heterozygous mutants thoroughly backcrossed to *w* ^Dah^ were mated to each other, and wild-type, heterozygous mutant, and homozygous mutant progeny were collected from the same bottles based on intensity of the transgenic eye colour marker. For details of rearing conditions and pre-experimental treatment, see Protocol S1. All lifespan studies were conducted in vials at 25 °C on a 12L:12D cycle at constant humidity. The flies were transferred to new vials three times per week and deaths were scored every day or every other day. Log-rank tests of survivorship curves were performed by using JMP IN statistical software (SAS Institute, http://www.sas.com).

### Molecular analysis of *Indy* mutants.

Authenticity of the P{lacW}*Indy^206^*, P{lacW}*Indy^302^,* and P{lacW}1085 insertions was confirmed in all genetic backgrounds by inverse PCR from genomic DNA followed by sequencing (unpublished data). Additionally, PCR reactions with P element–specific primer and primers specific to each insertion site in the genomic DNA were used (Figure S1). PCR for detection of *Wolbachia* was performed using primers wsp81F and wsp691R (kind gift from G. D. D. Hurst) as described before [47], and control reactions for DNA quality (dFoxo) were performed using primers FoxoEcoRIF (5′-GGGGAATTCGTTCAGTGCCGCCTCGGGACTTCC-3′) and FoxoNotI R

(5′-GATCGCGGCCGCGTCCTATCAAAGTAGAGGCGCAGT-3′). For expression analysis, RNA was extracted from 20 males per genotype and cDNA was prepared using standard Trizol methods (Invitrogen, http://www.invitrogen.com). Splice variant–specific PCR was performed from various cDNAs using the following 5′ primers in combination with common region primer IndyR-31 (5′-GTTTAGCAGCATAACAGGCAGACATA-3′): IndyRA-51 (5′-ATCGGACGAACCGGGCGTG-3′), IndyRB-51 (5′-GCAACATATTCATAAAAAGTGGTCTAGCC-3′), and IndyRC-51 (5′-CACTCGTTTTCATTCCAATTTTTGCGC-3′). The control primers for *Catalase (Cat)* were Cat-51 (5′-CGGCTTCCAATCAGTTGATTGACTAC-3′) and Cat-31 (5′-TCACATCCTGCAGCAGGATAGG-3′). *Catalase* was used as a control because it is the gene proximal to *Indy,* and we wanted to exclude the effect of *Indy* mutations of *Cat* expression. Northern hybridization was repeated twice using a probe specific to the common region of *Indy* (Figure 3A, grey box). The primers used to create the probe were IndyR-51 (5′-CGCCACTGGACATCAAAATGGAAAT-3′) and IndyR-31 (above). Loading was controlled by ribosomal protein rp49 probe that was amplified as above using primers rp49F (5′-AGCATACAGGCCCAAGATCG-3′) and rp49R (5′-CACCAGGAACTTCTTGAATCCGG-3′). Signals from northern blots were quantified by measuring the ^32^P-stimulated luminescence (PSL) using the FLA-2000 radioisotopic imaging system with Multi Gauge image analysis software (Fujifilm, http://www.fujifilm.com).

### C. elegans methodologies.

Lifespan studies: Bacteria-mediated RNA interference (RNAi) was used to inhibit gene function [61]. For the nonpreinduced method (Figure 7A and 7B), bacteria (E. coli) were grown for 14 h in liquid culture without IPTG, then seeded onto nematode growth medium plates containing 1mM IPTG and 50 μg/ml ampicillin. Seeded plates were allowed to dry for 48 h at room temperature. In the preinduced experiment (Figure 7C), preinduction with 0.4 mM IPTG was performed in the liquid culture 4 h before plating. The empty vector L4440 (pPD129) was used as a negative control. As a positive control for the efficacy of the RNAi treatment, we used a *daf-2* RNAi feeding strain previously shown to extend lifespan by ~80% [62]. The RNAi clones for *nac-2, nac-3,* and the control vector pPD129 were kindly provided by Y.-Y. Fei [34,35]. The *daf-2* RNAi clone was kindly provided by A. Dillin [62]. The presence of the correct inserts in each feeding vector was confirmed by DNA sequencing. A wild-type C. elegans strain N2 (Bristol) was provided by the *Caenorhabditis* Genetics Center (http://www.cbs.umn.edu/CGC). Lifespan measurements were performed at 22 °C on age-synchronous populations of nematodes as described previously [34].

RT-PCR methods: Eggs prepared from hypochlorite treatment were plated out onto the respective RNAi feeding bacteria, grown to the L4 stage, and harvested for RNA extraction. Four washes with M9 were used to remove residual bacteria. Total RNA was isolated using the Trizol reagent (Invitrogen). First-strand cDNA was generated from 2 μg of total RNA for each condition using reverse transcriptase priming with Oligo(dT)_12–18_ primer. cDNA was amplified using two pairs of PCR primers, one pair specific to either *ce*-*nac-2 or ce-nac-3* and a second set specific to *ama-1,* the internal control. Oligonucleotides were designed to cover exon/intron boundaries such that only cDNA would be amplified. Cycle numbers were optimised for each primer set to ensure the reaction was within the linear range and each reaction was terminated before reagents became limiting. The intensity of the RT-PCR bands were determined from the agarose gel using the Syngene imaging system with Genesnap and Genetools software (http://www.syngene.com). Levels of *ce*-*nac-2* and *ce-nac-3* were calculated as a relative intensity to the intensity of the *ama-1* RT-PCR product. The oligonucleotides used were: *ama-1* (5′-ATCTCGCAGGTTATCGCGTG-3′ and 5′-CGGTGAGGTCCATTCTGAAATC-3′), *ce-nac-2* (5′-TATTCACAAGAGATACCCCGAG-3′ and 5′-TCCCGATTTATCAACTCCTTCTG-3′), and *ce-nac-3* (5′-CAAATGGAGAACGTGGCCGTC-3′ and 5′-CGGAGCATCTCTCAAGAAGAAG-3′).

## Supporting Information

Figure S1 Authenticity of the *Indy* Mutant Lines Confirmed by PCR Analysis (102 KB PPT)

Figure S2 Lack of Longevity in *Indy^302^* Flies (72 KB PPT)

Protocol S1 Supporting Materials and Methods (28 KB DOC)

### Accession Numbers

National Center for Biotechnology information (NCBI) Entrez Gene ID numbers (http://www.ncbi.nlm.nih.gov/entrez) and UniProtKB/Swiss-Prot accession numbers (http://www.ebi.uniprot.org) for genes and proteins, respectively: *bonsai* (37587/Q8WTC1), *Cat* (40048/P17336), *daf-2* (175410/Q968Y9), *Indy* (40049/Q9VVT2), *nac-1* (181585/Q93655), *nac-2* (187898/P32739), *nac-3* (176429/Q21339), *sesB* (32007/Q26365), *tko* (31228/P10735), *w* (31271/P10090), and *wsp* (2738559/Q09TN6).

Allele-specific FlyBase ID numbers (http://flybase.bio.indiana.edu): *bonsai^1^* (FBal0097167), P{lacW}1085 (FBti0003775), P{lacW}*Indy^206^* (FBti0004258), P{lacW}*Indy^302^* (FBti0003781), *sesB^1^* (FBal0015434), *tko^25t^* (FBal0016812), and *w* ^1118^ (FBal0018186).

## Abbreviations

CS: Canton S
npi: non-per-induced; pi, pre-induced
RNAi: RNA interference
RT-PCR: reverse transcriptase PCR
SY: sugar and yeast based
TET: tetracycline
w^Dah^: white Dahomey: *wsp, Wolbachia surface protein*

## References

[pgen-0030095-b001] Kenyon C, Chang J, Gensch E, Rudner A, Tabtiang R (1993). A C. elegans mutant that lives twice as long as wild type. Nature.

[pgen-0030095-b002] Clancy DJ, Gems D, Harshman LG, Oldham S, Stocker H (2001). Extension of life-span by loss of CHICO, a *Drosophila* insulin receptor substrate protein. Science.

[pgen-0030095-b003] Tatar M, Kopelman A, Epstein D, Tu MP, Yin CM (2001). A mutant *Drosophila* insulin receptor homolog that extends life-span and impairs neuroendocrine function. Science.

[pgen-0030095-b004] Hwangbo DS, Gershman B, Tu MP, Palmer M, Tatar M (2004). *Drosophila* dFOXO controls lifespan and regulates insulin signalling in brain and fat body. Nature.

[pgen-0030095-b005] Giannakou ME, Goss M, Junger MA, Hafen E, Leevers SJ (2004). Long-lived *Drosophila* with overexpressed dFOXO in adult fat body. Science.

[pgen-0030095-b006] Vellai T, Takacs-Vellai K, Zhang Y, Kovacs AL, Orosz L (2003). Genetics: Influence of TOR kinase on lifespan in C. elegans. Nature.

[pgen-0030095-b007] Kapahi P, Zid BM, Harper T, Koslover D, Sapin V (2004). Regulation of lifespan in *Drosophila* by modulation of genes in the TOR signaling pathway. Curr Biol.

[pgen-0030095-b008] Powers RW, Kaeberlein M, Caldwell SD, Kennedy BK, Fields S (2006). Extension of chronological life span in yeast by decreased TOR pathway signaling. Genes Dev.

[pgen-0030095-b009] Wang MC, Bohmann D, Jasper H (2003). JNK signaling confers tolerance to oxidative stress and extends lifespan in *Drosophila*. Dev Cell.

[pgen-0030095-b010] Oh SW, Mukhopadhyay A, Svrzikapa N, Jiang F, Davis RJ (2005). JNK regulates lifespan in Caenorhabditis elegans by modulating nuclear translocation of forkhead transcription factor/DAF-16. Proc Natl Acad Sci U S A.

[pgen-0030095-b011] Wang MC, Bohmann D, Jasper H (2005). JNK extends life span and limits growth by antagonizing cellular and organism-wide responses to insulin signaling. Cell.

[pgen-0030095-b012] Mackay TF (2002). The nature of quantitative genetic variation for *Drosophila* longevity. Mech Ageing Dev.

[pgen-0030095-b013] Nuzhdin SV, Khazaeli AA, Curtsinger JW (2005). Survival analysis of life span quantitative trait loci in Drosophila melanogaster. Genetics.

[pgen-0030095-b014] Valenzuela RK, Forbes SN, Keim P, Service PM (2004). Quantitative trait loci affecting life span in replicated populations of Drosophila melanogaster. II. Response to selection. Genetics.

[pgen-0030095-b015] Forbes SN, Valenzuela RK, Keim P, Service PM (2004). Quantitative trait loci affecting life span in replicated populations of Drosophila melanogaster. I. Composite interval mapping. Genetics.

[pgen-0030095-b016] Geiger-Thornsberry GL, Mackay TF (2004). Quantitative trait loci affecting natural variation in Drosophila longevity. Mech Ageing Dev.

[pgen-0030095-b017] Swindell WR, Bouzat JL (2006). Inbreeding depression and male survivorship in *Drosophila:* Implications for senescence theory. Genetics.

[pgen-0030095-b018] Linnen C, Tatar M, Promislow DE (2001). Cultural artifacts: A comparison of senescence in natural, laboratory-adapted and artificially selected line of Drosophila melanogaster. Evol Ecol Res.

[pgen-0030095-b019] Spencer CC, Howell CE, Wright AR, Promislow DE (2003). Testing an “aging gene” in long-lived *Drosophila* strains: Increased longevity depends on sex and genetic background. Aging Cell.

[pgen-0030095-b020] Sun J, Tower J (1999). FLP recombinase-mediated induction of Cu/Zn-superoxide dismutase transgene expression can extend the life span of adult Drosophila melanogaster flies. Mol Cell Biol.

[pgen-0030095-b021] Rollmann SM, Magwire MM, Morgan TJ, Ozsoy ED, Yamamoto A (2006). Pleiotropic fitness effects of the Tre1-Gr5a region in Drosophila melanogaster. Nat Genet.

[pgen-0030095-b022] Miller RA, Harper JM, Dysko RC, Durkee SJ, Austad SN (2002). Longer life spans and delayed maturation in wild-derived mice. Exp Biol Med (Maywood).

[pgen-0030095-b023] Harper JM, Durkee SJ, Dysko RC, Austad SN, Miller RA (2006). Genetic modulation of hormone levels and life span in hybrids between laboratory and wild-derived mice. J Gerontol A Biol Sci Med Sci.

[pgen-0030095-b024] Sgrò CM, Partridge L (2000). Evolutionary responses of the life history of wild-caught *Drosophila melanogaster* to two standard methods of laboratory culture. Amer Natur.

[pgen-0030095-b025] Min KT, Benzer S (1997). *Wolbachia,* normally a symbiont of *Drosophila,* can be virulent, causing degeneration and early death. Proc Natl Acad Sci U S A.

[pgen-0030095-b026] Fry AJ, Rand DM (2002). *Wolbachia* interactions that determine Drosophila melanogaster survival. Evolution Int J Org Evolution.

[pgen-0030095-b027] Fry AJ, Palmer MR, Rand DM (2004). Variable fitness effects of *Wolbachia* infection in Drosophila melanogaster. Heredity.

[pgen-0030095-b028] Driver C, Georgiou A, Georgiou G (2004). The contribution by mitochondrially induced oxidative damage to aging in Drosophila melanogaster. Biogerontology.

[pgen-0030095-b029] Dean MD (2006). A *Wolbachia*-associated fitness benefit depends on genetic background in Drosophila simulans. Proc Biol Sci.

[pgen-0030095-b030] Clark ME, Anderson CL, Cande J, Karr TL (2005). Widespread prevalence of *Wolbachia* in laboratory stocks and the implications for *Drosophila* research. Genetics.

[pgen-0030095-b031] Rogina B, Reenan RA, Nilsen SP, Helfand SL (2000). Extended life-span conferred by cotransporter gene mutations in *Drosophila*. Science.

[pgen-0030095-b032] Knauf F, Mohebbi N, Teichert C, Herold D, Rogina B (2006). The life-extending gene *Indy* encodes an exchanger for Krebs-cycle intermediates. Biochem J.

[pgen-0030095-b033] Knauf F, Rogina B, Jiang Z, Aronson PS, Helfand SL (2002). Functional characterization and immunolocalization of the transporter encoded by the life-extending gene *Indy*. Proc Natl Acad Sci U S A.

[pgen-0030095-b034] Fei YJ, Inoue K, Ganapathy V (2003). Structural and functional characteristics of two sodium-coupled dicarboxylate transporters (ceNaDC1 and ceNaDC2) from Caenorhabditis elegans and their relevance to life span. J Biol Chem.

[pgen-0030095-b035] Fei YJ, Liu JC, Inoue K, Zhuang L, Miyake K (2004). Relevance of NAC-2, an Na+-coupled citrate transporter, to life span, body size and fat content in Caenorhabditis elegans. Biochem J.

[pgen-0030095-b036] Ishii N, Takahashi K, Tomita S, Keino T, Honda S (1990). A methyl viologen-sensitive mutant of the nematode Caenorhabditis elegans. Mutat Res.

[pgen-0030095-b037] Kondo M, Senoo-Matsuda N, Yanase S, Ishii T, Hartman PS (2005). Effect of oxidative stress on translocation of DAF-16 in oxygen-sensitive mutants, mev-1 and gas-1 of Caenorhabditis elegans. Mech Ageing Dev.

[pgen-0030095-b038] Feng J, Bussiere F, Hekimi S (2001). Mitochondrial electron transport is a key determinant of life span in Caenorhabditis elegans. Dev Cell.

[pgen-0030095-b039] Dillin A, Hsu AL, Arantes-Oliveira N, Lehrer-Graiwer J, Hsin H (2002). Rates of behavior and aging specified by mitochondrial function during development. Science.

[pgen-0030095-b040] Lee SS, Lee RY, Fraser AG, Kamath RS, Ahringer J (2003). A systematic RNAi screen identifies a critical role for mitochondria in C. elegans longevity. Nat Genet.

[pgen-0030095-b041] Judd BH, Shen MW, Kaufman TC (1972). The anatomy and function of a segment of the X chromosome of Drosophila melanogaster. Genetics.

[pgen-0030095-b042] Royden CS, Pirrotta V, Jan LY (1987). The tko locus, site of a behavioral mutation in *D. melanogaster,* codes for a protein homologous to prokaryotic ribosomal protein S12. Cell.

[pgen-0030095-b043] Toivonen JM, O'Dell KM, Petit N, Irvine SC, Knight GK (2001). Technical knockout, a *Drosophila* model of mitochondrial deafness. Genetics.

[pgen-0030095-b044] Galloni M, Edgar BA (1999). Cell-autonomous and non-autonomous growth-defective mutants of Drosophila melanogaster. Development.

[pgen-0030095-b045] Baldal EA, Baktawar W, Brakefield PM, Zwaan BJ (2006). Methuselah life history in a variety of conditions, implications for the use of mutants in longevity research. Exp Gerontol.

[pgen-0030095-b046] Grumbling G, Strelets V (2006). FlyBase: Anatomical data, images and queries. Nucleic Acids Res.

[pgen-0030095-b047] Zhou W, Rousset F, O'Neil S (1998). Phylogeny and PCR-based classification of *Wolbachia* strains using wsp gene sequences. Proc Biol Sci.

[pgen-0030095-b048] Braig HR, Zhou W, Dobson SL, O'Neill SL (1998). Cloning and characterization of a gene encoding the major surface protein of the bacterial endosymbiont Wolbachia pipientis. J Bacteriol.

[pgen-0030095-b049] Bauer JH, Goupil S, Garber GB, Helfand SL (2004). An accelerated assay for the identification of lifespan-extending interventions in Drosophila melanogaster. Proc Natl Acad Sci U S A.

[pgen-0030095-b050] Helfand SL, Blake KJ, Rogina B, Stracks MD, Centurion A (1995). Temporal patterns of gene expression in the antenna of the adult Drosophila melanogaster. Genetics.

[pgen-0030095-b051] Helfand SL, Naprta B (1996). The expression of a reporter protein, beta-galactosidase, is preserved during maturation and aging in some cells of the adult Drosophila melanogaster. Mech Dev.

[pgen-0030095-b052] Khazaeli AA, Van Voorhies W, Curtsinger JW (2005). Longevity and metabolism in *Drosophila melanogaster:* genetic correlations between life span and age-specific metabolic rate in populations artificially selected for long life. Genetics.

[pgen-0030095-b053] Gargano JW, Martin I, Bhandari P, Grotewiel MS (2005). Rapid iterative negative geotaxis (RING): A new method for assessing age-related locomotor decline in *Drosophila*. Exp Gerontol.

[pgen-0030095-b054] Martin I, Grotewiel MS (2006). Distinct genetic influences on locomotor senescence in *Drosophila* revealed by a series of metrical analyses. Exp Gerontol.

[pgen-0030095-b055] Rand DM, Fry A, Sheldahl L (2006). Nuclear-mitochondrial epistasis and Drosophila aging: Introgression of Drosophila simulans mtDNA modifies longevity in D. melanogaster nuclear backgrounds. Genetics.

[pgen-0030095-b056] Partridge L, Piper MD, Mair W (2005). Dietary restriction in *Drosophila*. Mech Ageing Dev.

[pgen-0030095-b057] Piper MD, Skorupa D, Partridge L (2005). Diet, metabolism and lifespan in *Drosophila*. Exp Gerontol.

[pgen-0030095-b058] Rogina B, Helfand SL, Frankel S (2002). Longevity regulation by *Drosophila* Rpd3 deacetylase and caloric restriction. Science.

[pgen-0030095-b059] Rogina B, Helfand SL (2004). Sir2 mediates longevity in the fly through a pathway related to calorie restriction. Proc Natl Acad Sci U S A.

[pgen-0030095-b060] Boynton S, Tully T (1992). latheo, a new gene involved in associative learning and memory in *Drosophila melanogaster,* identified from P element mutagenesis. Genetics.

[pgen-0030095-b061] Kamath RS, Martinez-Campos M, Zipperlen P, Fraser AG, Ahringer J (2001). Effectiveness of specific RNA-mediated interference through ingested double-stranded RNA in Caenorhabditis elegans. Genome Biol.

[pgen-0030095-b062] Dillin A, Crawford DK, Kenyon C (2002). Timing requirements for insulin/IGF-1 signaling in C. elegans. Science.

