# Supplementary material for: No Influence of Indy on Lifespan in Drosophila after Correction for Genetic and Cytoplasmic Background Effects
Source: PLoS Genet. 2007 Jun 15;3(6):e95. doi: 10.1371/journal.pgen.0030095 (PMC1892600; doi:10.1371/journal.pgen.0030095)
Supplement: Supplementary file 1 [file pgen.0030095.sd001.doc]

**Supporting Materials and Methods:**

**Fly culture media.** The two types of food used in the lifespan experiments (except virgin females, see below) were SY (50g sucrose, 100g brewer's yeast, 15g agar, 3g Nipagin^®^ M and 3ml propionic acid per litre) and corn meal food (124g sucrose, 31g yeast, 53g cornmeal, 8.9g agar and 2.6g Nipagin^®^ M per litre). The former is the food medium most commonly used in our laboratory whereas the latter was prepared as according to the method given in Rogina et al. (2000). The brewer's yeast used in the food (cat #: 903312) was supplied by MP Biomedicals, London, UK, the corn meal was obtained from T.P. Drewitt, London UK, the sucrose was from Tate & Lyle Sugars, London, UK and the agar and propionic acid from Sigma, Dorset, UK. Nipagin^®^ M (methyl 4-hydroxybenzoate, Clariant UK Ltd, Pontypridd, UK) was added in a solution containing 100 g/L Nipagin^®^ M in 95% ethanol. For virgin female experiments another variation of SY food was used (100g sucrose, 100g baker's yeast (T.P. Drewitt, London, UK), 20g agar, 3g Nipagin^®^ M and 3ml propionic acid per litre).

**Generation of experimental flies.** To generate experimental flies, age-matched parents were mated in 2-4 cages containing grape juice agar plates with live yeast paste. After two days of acclimatization, eggs laid during a six-hour window were collected, and the same volume of embryos was transferred to each rearing bottle for a rearing density of 300 – 350 flies per bottle. For experiments with mated flies, newly eclosed flies were transferred to new bottles without anaesthesia and left for 48 hours to mate. Sexes were separated by brief CO_2_ exposure and the flies were transferred into experimental vials, ten flies per vial. When both sexes were kept in the same vial, five females and five males were placed in the same vial. For experiments with virgins, females were collected on ice (to avoid early lethality associated with CO_2_ -exposure) within six hours after eclosion.

**Transfer of chromosome X and cytoplasmic constituents from Indy-206 to wDah background (data in figure 6).** In the following crossing scheme, +^CS^ denotes a non-recombined chromosome derived from CS-Indy^206^ line (referred as CS for clarity), +^WD^ denotes a a non-recombined chromosome derived from *white* Dahomey line (WD). Chromosomes X/Y, 2 and 3 are separated with semi columns. +^CS/WD^ signifies chromosomes that are recombination products of those derived from CS-Indy^206^ and *white* Dahomey. In each cross, genotype on the left refers to female flies and genotype on the right refers to male flies.

*1) Creation of multiple balancer stock in w^Dah^ genetic background (#).*Balancers for chromosomes X (FM7a), 2 (CyO), and 3 (TM6B,Tb) were crossed with *white* Dahomey (WD) to create a stock where all non-balancers chromosomes (and cytoplasmic constituents) are +^WD^. Various combinations of balancers required in the next steps were collected from this **(#)** stock.

1A) +^WD^/+^WD^; +^WD^/+^WD^; +^WD^/+^WD^	**X**	FM7a/Y; CyO/Sp: TM6B,Tb/MKRS,Sb

1B) +^WD^/FM7a; +^WD^/CyO; +^WD^/TM6B,Tb	**X**	+^WD^/Y^WD^; +^WD^/+^WD^; +^WD^/+^WD^

1C) +^WD^/FM7a; +^WD^/CyO; +^WD^/TM6B,Tb	**X**	FM7a/Y^WD^; +^WD^/CyO; +^WD^/TM6B,Tb

*2) Generation of a stock where Chromosome X is from CS, everything else is from WD.*

2A) +^CS^/+^CS^; +^CS^/+^CS^; I-206^CS^**/**I-206^CS^	**X**	FM7a/Y^WD^; +^WD^/+^WD^; +^WD^/+^WD^ **(#)**

2B) +^CS^/FM7a; +^CS^/+^WD^; I-206^CS^/+^WD^	**X**	FM7a/Y^WD^; +^WD^/CyO: +^WD^/TM6B,Tb **(#)**

2C) +^WD^/FM7a; +^WD^/+^WD^; +^WD^/+^WD^ **(#)**	**X**	+^CS^/Y^WD^; +^CS/WD^/CyO; I-206^CS/WD^/TM6B,Tb

2D) +^CS^/FM7a; +^WD^/CyO; +^WD^/TM6B,Tb	**X**	+^CS^/ Y^WD^; +^WD^/CyO; +^WD^/TM6B,Tb

*3 ) Generation of a stock where cytoplasm is from CS, everything else is from WD.*

3A) +^CS^/+^CS^; +^CS^/+^CS^; I-206^CS^**/**I-206^CS^	**X**	FM7a/Y^WD^; +^WD^/+^WD^; +^WD^/+^WD^ **(#)**

3B) +^CS^/FM7a; +^CS^/+^WD^; I-206^CS^/+^WD^	**X**	+^WD^/Y^WD^; +^WD^/CyO; +^WD^/TM6B,Tb **(#)**

3C) +^WD^/FM7a; +^CS/WD^/CyO; I-206^CS/WD^/TM6B,Tb	**X**	+^WD^/ Y^WD^; +^WD^/+^WD^; +^WD^/+^WD^ **(#)**

3D) +^WD^/FM7a; +^WD^/CyO; +^WD^/TM6B,Tb	**X**	+^WD^/ Y^WD^; +^WD^/CyO; +^WD^/TM6B,Tb

Non-balanced flies from crosses 2D and 3D were collected to establish the stocks. Females from these lines were crossed with w^Dah^ males to get experimental male flies (shown in figure 6).
